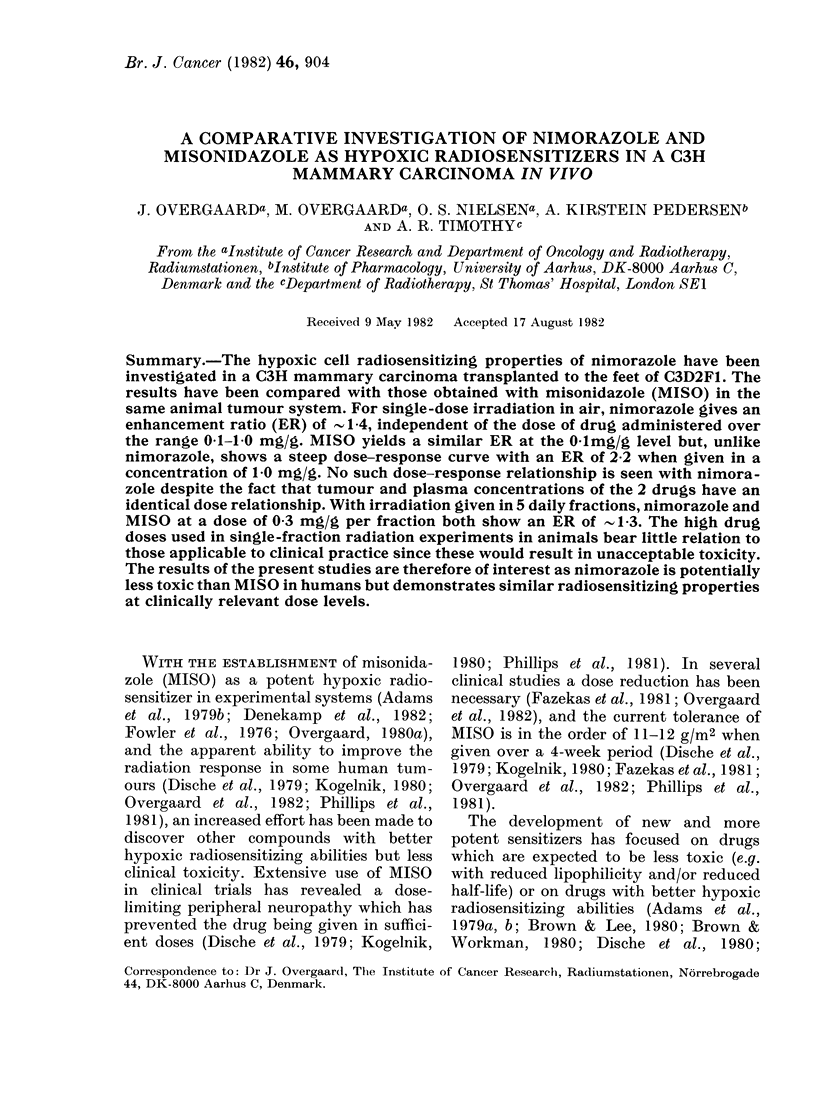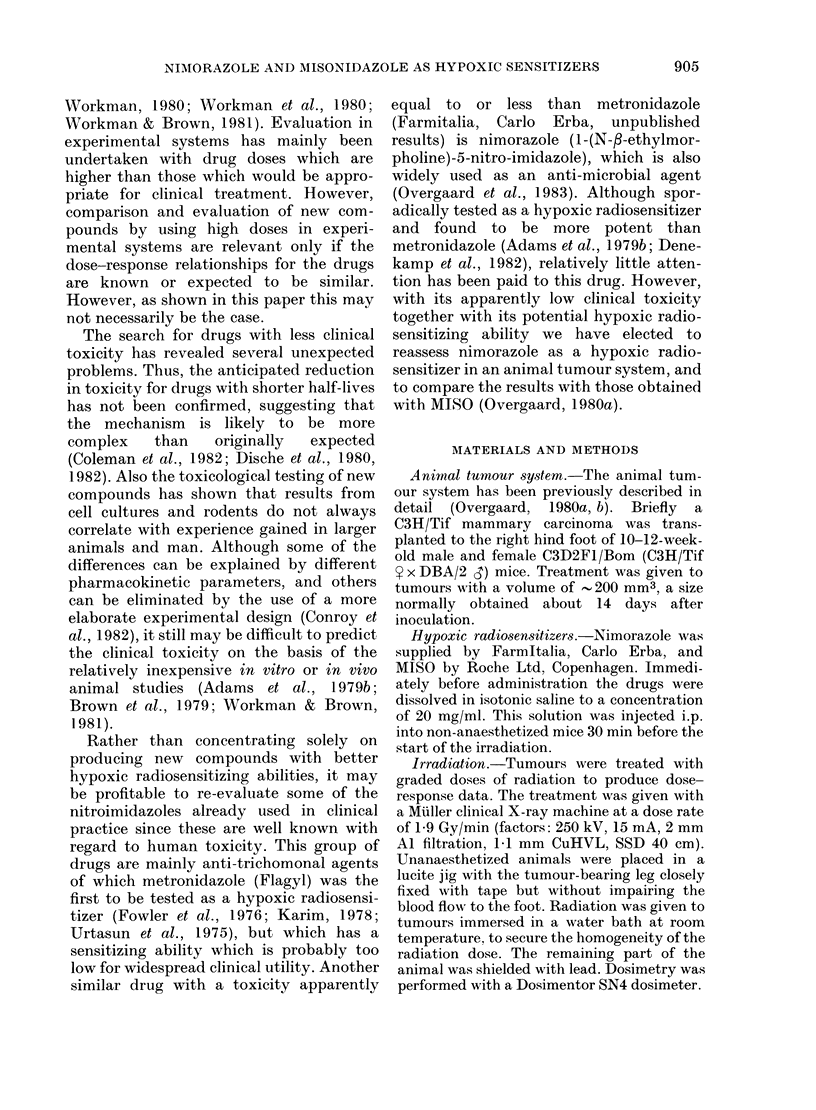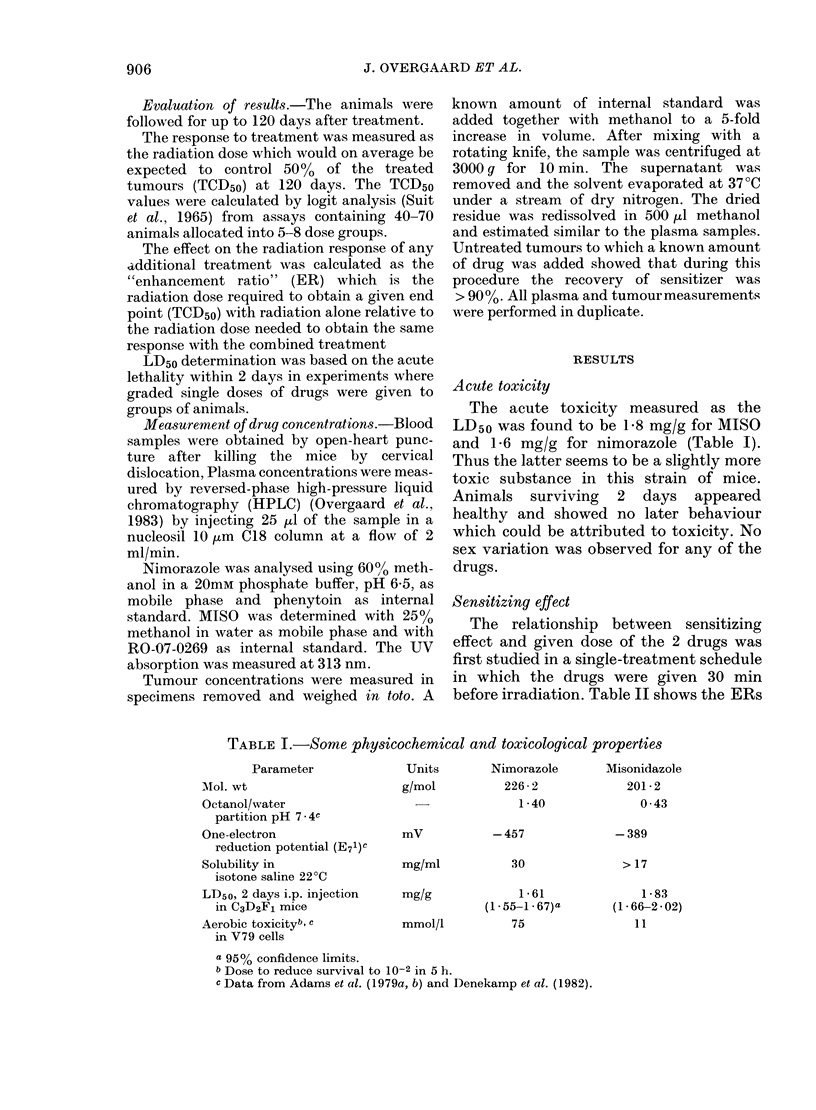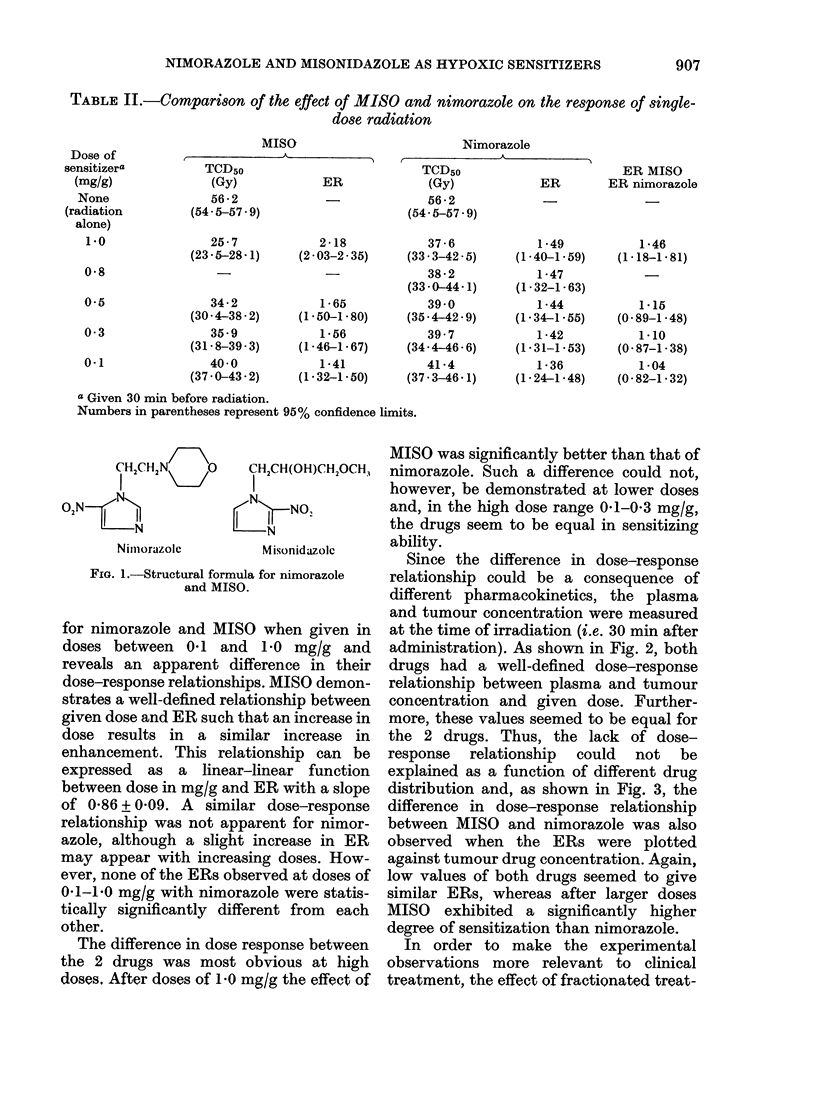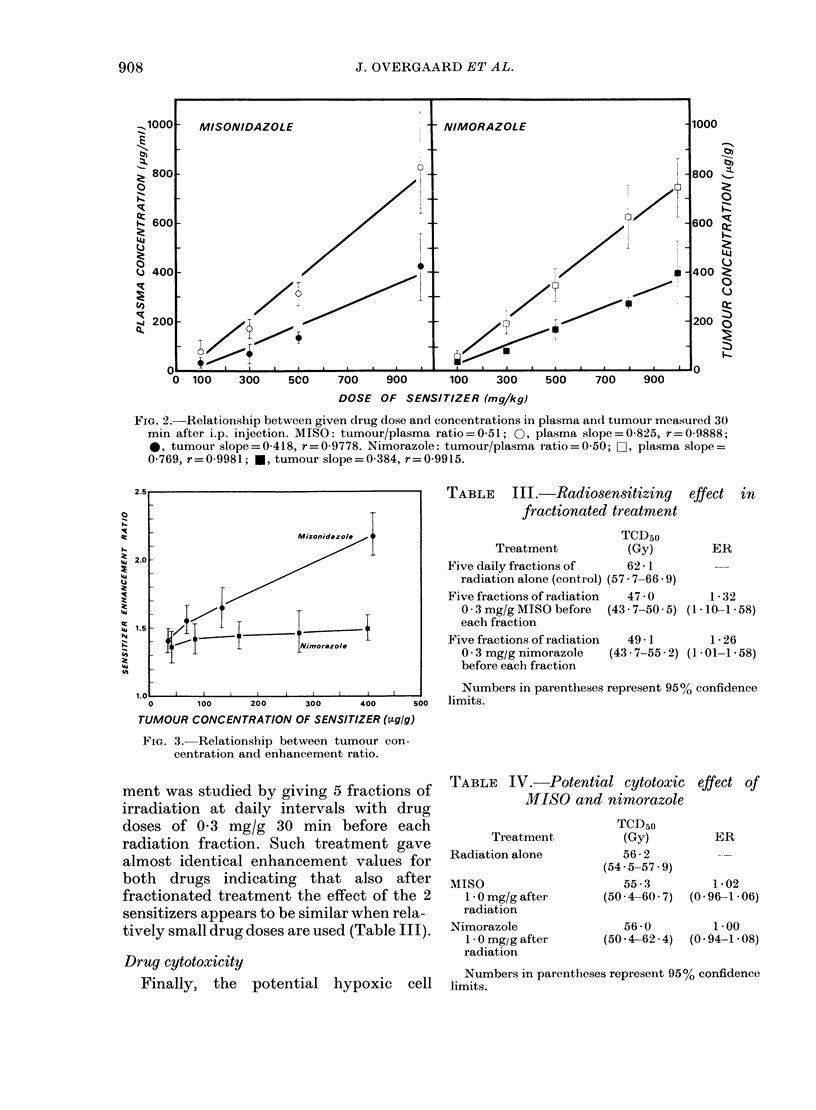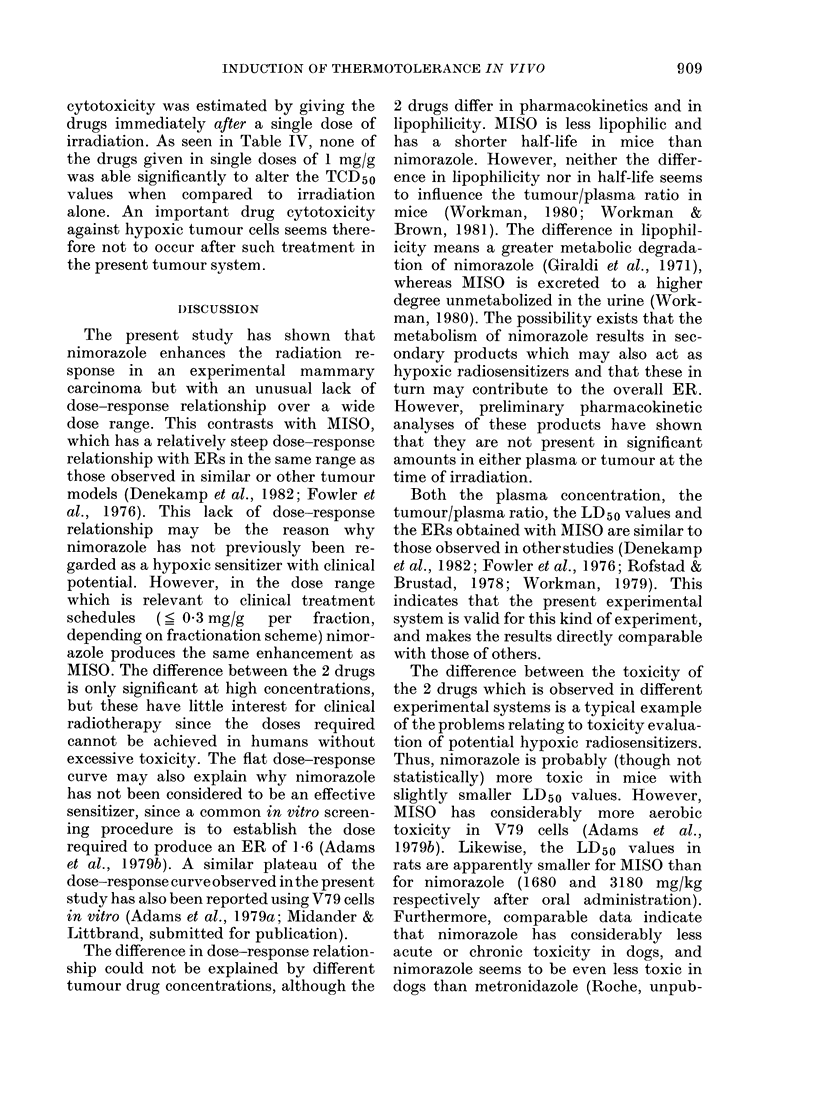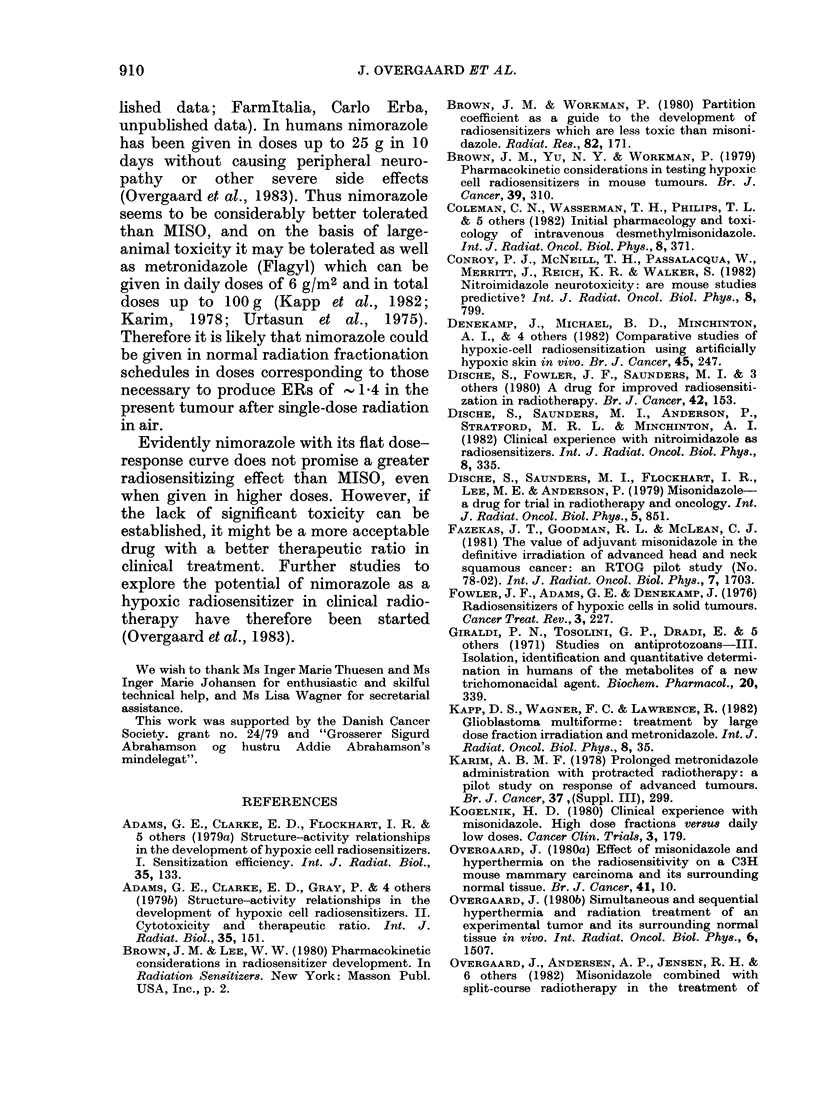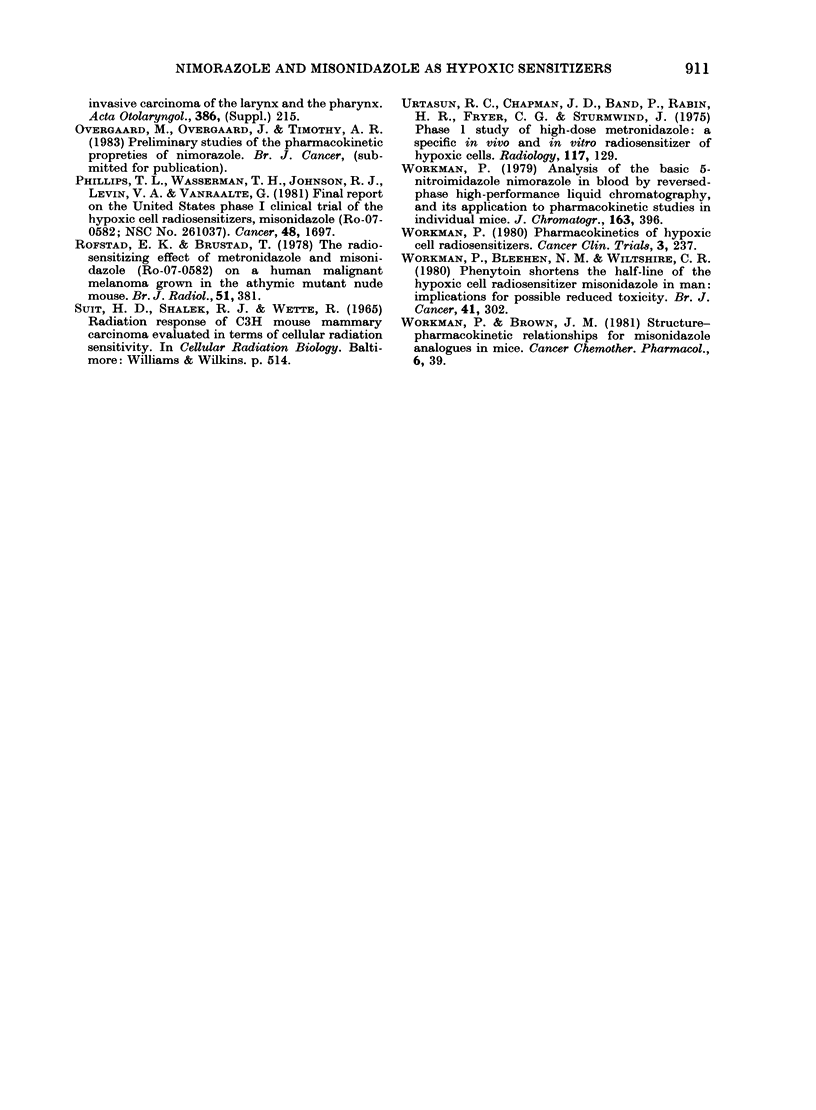# A comparative investigation of nimorazole and misonidazole as hypoxic radiosensitizers in a C3H mammary carcinoma in vivo.

**DOI:** 10.1038/bjc.1982.300

**Published:** 1982-12

**Authors:** J. Overgaard, M. Overgaard, O. S. Nielsen, A. K. Pedersen, A. R. Timothy

## Abstract

The hypoxic cell radiosensitizing properties of nimorazole have been investigated in a C3H mammary carcinoma transplanted to the feet of C3D2F1. The results have been compared with those obtained with misonidazole (MISO) in the same animal tumour system. For single-dose irradiation in air, nimorazole gives an enhancement ratio (ER) of approximately 1.4, independent of the dose of drug administered over the range 0.1-1.0 mg/g. MISO yields a similar ER at the 0.1 mg/g level but, unlike nimorazole, shows a steep dose-response curve with an ER of 2.2 when given in a concentration of 1.0 mg/g. No such dose-response relationship is seen with nimorazole despite the fact that tumour and plasma concentrations of the 2 drugs have an identical dose relationship. With irradiation given in 5 daily fractions, nimorazole and MISO at a dose of 0.3 mg/g per fraction both show an ER of approximately 1.3. The high drug doses used in single-fraction radiation experiments in animals bear little relation to those applicable to clinical practice since these would result in unacceptable toxicity. The results of the present studies are therefore of interest as nimorazole is potentially less toxic than MISO in humans but demonstrates similar radiosensitizing properties at clinically relevant dose levels.


*Br. J. Cancer* (1982) **46**, 904

### A COMPARATIVE INVESTIGATION OF NIMORAZOLE AND MISONIDAZOLE AS HYPOXIC RADIOSENSITIZERS IN A C3H MAMMARY CARCINOMA *IN VIVO*

J. OVERGAARD[a], M. OVERGAARD[a], O. S. NIELSEN[a], A. KIRSTEIN PEDERSEN[b] AND A. R. TIMOTHY[c]

*From the [a]Institute of Cancer Research and Department of Oncology and Radiotherapy, Radiumstationen, [b]Institute of Pharmacology, University of Aarhus, DK-8000 Aarhus C, Denmark and the [c]Department of Radiotherapy, St Thomas' Hospital, London SE1*

Received 9 May 1982 Accepted 17 August 1982

**Summary.—The hypoxic cell radiosensitizing properties of nimorazole have been investigated in a C3H mammary carcinoma transplanted to the feet of C3D2F1. The results have been compared with those obtained with misonidazole (MISO) in the same animal tumour system. For single-dose irradiation in air, nimorazole gives an enhancement ratio (ER) of ~1·4, independent of the dose of drug administered over the range 0·1–1·0 mg/g. MISO yields a similar ER at the 0·1mg/g level but, unlike nimorazole, shows a steep dose–response curve with an ER of 2·2 when given in a concentration of 1·0 mg/g. No such dose–response relationship is seen with nimorazole despite the fact that tumour and plasma concentrations of the 2 drugs have an identical dose relationship. With irradiation given in 5 daily fractions, nimorazole and MISO at a dose of 0·3 mg/g per fraction both show an ER of ~1·3. The high drug doses used in single-fraction radiation experiments in animals bear little relation to those applicable to clinical practice since these would result in unacceptable toxicity. The results of the present studies are therefore of interest as nimorazole is potentially less toxic than MISO in humans but demonstrates similar radiosensitizing properties at clinically relevant dose levels.**

WITH THE ESTABLISHMENT of misonidazole (MISO) as a potent hypoxic radiosensitizer in experimental systems (Adams *et al.*, 1979*b*; Denekamp *et al.*, 1982; Fowler *et al.*, 1976; Overgaard, 1980*a*), and the apparent ability to improve the radiation response in some human tumours (Dische *et al.*, 1979; Kogelnik, 1980; Overgaard *et al.*, 1982; Phillips *et al.*, 1981), an increased effort has been made to discover other compounds with better hypoxic radiosensitizing abilities but less clinical toxicity. Extensive use of MISO in clinical trials has revealed a dose-limiting peripheral neuropathy which has prevented the drug being given in sufficient doses (Dische *et al.*, 1979; Kogelnik, 1980; Phillips *et al.*, 1981). In several clinical studies a dose reduction has been necessary (Fazekas *et al.*, 1981; Overgaard *et al.*, 1982), and the current tolerance of MISO is in the order of 11–12 g/m$^2$ when given over a 4-week period (Dische *et al.*, 1979; Kogelnik, 1980; Fazekas *et al.*, 1981; Overgaard *et al.*, 1982; Phillips *et al.*, 1981).

The development of new and more potent sensitizers has focused on drugs which are expected to be less toxic (*e.g.* with reduced lipophilicity and/or reduced half-life) or on drugs with better hypoxic radiosensitizing abilities (Adams *et al.*, 1979*a*, *b*; Brown & Lee, 1980; Brown & Workman, 1980; Dische *et al.*, 1980;

Correspondence to: Dr J. Overgaard, The Institute of Cancer Research, Radiumstationen, Nörrebrogade 44, DK-8000 Aarhus C, Denmark.

Workman, 1980; Workman *et al.*, 1980; Workman & Brown, 1981). Evaluation in experimental systems has mainly been undertaken with drug doses which are higher than those which would be appropriate for clinical treatment. However, comparison and evaluation of new compounds by using high doses in experimental systems are relevant only if the dose–response relationships for the drugs are known or expected to be similar. However, as shown in this paper this may not necessarily be the case.

The search for drugs with less clinical toxicity has revealed several unexpected problems. Thus, the anticipated reduction in toxicity for drugs with shorter half-lives has not been confirmed, suggesting that the mechanism is likely to be more complex than originally expected (Coleman *et al.*, 1982; Dische *et al.*, 1980, 1982). Also the toxicological testing of new compounds has shown that results from cell cultures and rodents do not always correlate with experience gained in larger animals and man. Although some of the differences can be explained by different pharmacokinetic parameters, and others can be eliminated by the use of a more elaborate experimental design (Conroy *et al.*, 1982), it still may be difficult to predict the clinical toxicity on the basis of the relatively inexpensive *in vitro* or *in vivo* animal studies (Adams *et al.*, 1979*b*; Brown *et al.*, 1979; Workman & Brown, 1981).

Rather than concentrating solely on producing new compounds with better hypoxic radiosensitizing abilities, it may be profitable to re-evaluate some of the nitroimidazoles already used in clinical practice since these are well known with regard to human toxicity. This group of drugs are mainly anti-trichomonal agents of which metronidazole (Flagyl) was the first to be tested as a hypoxic radiosensitizer (Fowler *et al.*, 1976; Karim, 1978; Urtasun *et al.*, 1975), but which has a sensitizing ability which is probably too low for widespread clinical utility. Another similar drug with a toxicity apparently equal to or less than metronidazole (Farmitalia, Carlo Erba, unpublished results) is nimorazole (1-(N-β-ethylmorpholine)-5-nitro-imidazole), which is also widely used as an anti-microbial agent (Overgaard *et al.*, 1983). Although sporadically tested as a hypoxic radiosensitizer and found to be more potent than metronidazole (Adams *et al.*, 1979*b*; Denekamp *et al.*, 1982), relatively little attention has been paid to this drug. However, with its apparently low clinical toxicity together with its potential hypoxic radiosensitizing ability we have elected to reassess nimorazole as a hypoxic radiosensitizer in an animal tumour system, and to compare the results with those obtained with MISO (Overgaard, 1980*a*).

### MATERIALS AND METHODS

*Animal tumour system.*—The animal tumour system has been previously described in detail (Overgaard, 1980*a*, *b*). Briefly a C3H/Tif mammary carcinoma was transplanted to the right hind foot of 10–12-week-old male and female C3D2F1/Bom (C3H/Tif ♀ × DBA/2 ♂) mice. Treatment was given to tumours with a volume of ~200 mm³, a size normally obtained about 14 days after inoculation.

*Hypoxic radiosensitizers.*—Nimorazole was supplied by FarmItalia, Carlo Erba, and MISO by Roche Ltd, Copenhagen. Immediately before administration the drugs were dissolved in isotonic saline to a concentration of 20 mg/ml. This solution was injected i.p. into non-anaesthetized mice 30 min before the start of the irradiation.

*Irradiation.*—Tumours were treated with graded doses of radiation to produce dose–response data. The treatment was given with a Müller clinical X-ray machine at a dose rate of 1·9 Gy/min (factors: 250 kV, 15 mA, 2 mm Al filtration, 1·1 mm CuHVL, SSD 40 cm). Unanaesthetized animals were placed in a lucite jig with the tumour-bearing leg closely fixed with tape but without impairing the blood flow to the foot. Radiation was given to tumours immersed in a water bath at room temperature, to secure the homogeneity of the radiation dose. The remaining part of the animal was shielded with lead. Dosimetry was performed with a Dosimentor SN4 dosimeter.

*Evaluation of results.*—The animals were followed for up to 120 days after treatment.

The response to treatment was measured as the radiation dose which would on average be expected to control 50% of the treated tumours ($TCD_{50}$) at 120 days. The $TCD_{50}$ values were calculated by logit analysis (Suit *et al.*, 1965) from assays containing 40–70 animals allocated into 5–8 dose groups.

The effect on the radiation response of any additional treatment was calculated as the "enhancement ratio" (ER) which is the radiation dose required to obtain a given end point ($TCD_{50}$) with radiation alone relative to the radiation dose needed to obtain the same response with the combined treatment

$LD_{50}$ determination was based on the acute lethality within 2 days in experiments where graded single doses of drugs were given to groups of animals.

*Measurement of drug concentrations.*—Blood samples were obtained by open-heart puncture after killing the mice by cervical dislocation, Plasma concentrations were measured by reversed-phase high-pressure liquid chromatography (HPLC) (Overgaard *et al.*, 1983) by injecting 25 $\mu$l of the sample in a nucleosil 10 $\mu$m C18 column at a flow of 2 ml/min.

Nimorazole was analysed using 60% methanol in a 20mM phosphate buffer, pH 6·5, as mobile phase and phenytoin as internal standard. MISO was determined with 25% methanol in water as mobile phase and with RO-07-0269 as internal standard. The UV absorption was measured at 313 nm.

Tumour concentrations were measured in specimens removed and weighed *in toto*. A known amount of internal standard was added together with methanol to a 5-fold increase in volume. After mixing with a rotating knife, the sample was centrifuged at 3000 *g* for 10 min. The supernatant was removed and the solvent evaporated at 37 °C under a stream of dry nitrogen. The dried residue was redissolved in 500 $\mu$l methanol and estimated similar to the plasma samples. Untreated tumours to which a known amount of drug was added showed that during this procedure the recovery of sensitizer was > 90%. All plasma and tumour measurements were performed in duplicate.

### RESULTS

### *Acute toxicity*

The acute toxicity measured as the $LD_{50}$ was found to be 1·8 mg/g for MISO and 1·6 mg/g for nimorazole (Table I). Thus the latter seems to be a slightly more toxic substance in this strain of mice. Animals surviving 2 days appeared healthy and showed no later behaviour which could be attributed to toxicity. No sex variation was observed for any of the drugs.

### *Sensitizing effect*

The relationship between sensitizing effect and given dose of the 2 drugs was first studied in a single-treatment schedule in which the drugs were given 30 min before irradiation. Table II shows the ERs

TABLE I.—*Some physicochemical and toxicological properties*

| Parameter | Units | Nimorazole | Misonidazole |
|---|---|---|---|
| Mol. wt | g/mol | 226·2 | 201·2 |
| Octanol/water partition pH 7·4[c] | — | 1·40 | 0·43 |
| One-electron reduction potential ($E_7^1$)[c] | mV | −457 | −389 |
| Solubility in isotone saline 22 °C | mg/ml | 30 | > 17 |
| $LD_{50}$, 2 days i.p. injection in $C_3D_2F_1$ mice | mg/g | 1·61 (1·55–1·67)[a] | 1·83 (1·66–2·02) |
| Aerobic toxicity[b, c] in V79 cells | mmol/l | 75 | 11 |

[a] 95% confidence limits.
[b] Dose to reduce survival to $10^{-2}$ in 5 h.
[c] Data from Adams *et al.* (1979*a, b*) and Denekamp *et al.* (1982).

TABLE II.—*Comparison of the effect of MISO and nimorazole on the response of single-dose radiation*

| Dose of sensitizer[a] (mg/g) | MISO TCD50 (Gy) | MISO ER | Nimorazole TCD50 (Gy) | Nimorazole ER | ER MISO / ER nimorazole |
|---|---|---|---|---|---|
| None (radiation alone) | 56·2 (54·5–57·9) | — | 56·2 (54·5–57·9) | — | — |
| 1·0 | 25·7 (23·5–28·1) | 2·18 (2·03–2·35) | 37·6 (33·3–42·5) | 1·49 (1·40–1·59) | 1·46 (1·18–1·81) |
| 0·8 | — | — | 38·2 (33·0–44·1) | 1·47 (1·32–1·63) | — |
| 0·5 | 34·2 (30·4–38·2) | 1·65 (1·50–1·80) | 39·0 (35·4–42·9) | 1·44 (1·34–1·55) | 1·15 (0·89–1·48) |
| 0·3 | 35·9 (31·8–39·3) | 1·56 (1·46–1·67) | 39·7 (34·4–46·6) | 1·42 (1·31–1·53) | 1·10 (0·87–1·38) |
| 0·1 | 40·0 (37·0–43·2) | 1·41 (1·32–1·50) | 41·4 (37·3–46·1) | 1·36 (1·24–1·48) | 1·04 (0·82–1·32) |

[a] Given 30 min before radiation.
Numbers in parentheses represent 95% confidence limits.

FIG. 1.—Structural formula for nimorazole and MISO.

for nimorazole and MISO when given in doses between 0·1 and 1·0 mg/g and reveals an apparent difference in their dose–response relationships. MISO demonstrates a well-defined relationship between given dose and ER such that an increase in dose results in a similar increase in enhancement. This relationship can be expressed as a linear–linear function between dose in mg/g and ER with a slope of $0{\cdot}86 \pm 0{\cdot}09$. A similar dose–response relationship was not apparent for nimorazole, although a slight increase in ER may appear with increasing doses. However, none of the ERs observed at doses of 0·1–1·0 mg/g with nimorazole were statistically significantly different from each other.

The difference in dose response between the 2 drugs was most obvious at high doses. After doses of 1·0 mg/g the effect of MISO was significantly better than that of nimorazole. Such a difference could not, however, be demonstrated at lower doses and, in the high dose range 0·1–0·3 mg/g, the drugs seem to be equal in sensitizing ability.

Since the difference in dose–response relationship could be a consequence of different pharmacokinetics, the plasma and tumour concentration were measured at the time of irradiation (*i.e.* 30 min after administration). As shown in Fig. 2, both drugs had a well-defined dose–response relationship between plasma and tumour concentration and given dose. Furthermore, these values seemed to be equal for the 2 drugs. Thus, the lack of dose–response relationship could not be explained as a function of different drug distribution and, as shown in Fig. 3, the difference in dose–response relationship between MISO and nimorazole was also observed when the ERs were plotted against tumour drug concentration. Again, low values of both drugs seemed to give similar ERs, whereas after larger doses MISO exhibited a significantly higher degree of sensitization than nimorazole.

In order to make the experimental observations more relevant to clinical treatment, the effect of fractionated treat-

FIG. 2.—Relationship between given drug dose and concentrations in plasma and tumour measured 30 min after i.p. injection. MISO: tumour/plasma ratio = 0·51; ○, plasma slope = 0·825, $r = 0{\cdot}9888$; ●, tumour slope = 0·418, $r = 0{\cdot}9778$. Nimorazole: tumour/plasma ratio = 0·50; □, plasma slope = 0·769, $r = 0{\cdot}9981$; ■, tumour slope = 0·384, $r = 0{\cdot}9915$.

FIG. 3.—Relationship between tumour concentration and enhancement ratio.

ment was studied by giving 5 fractions of irradiation at daily intervals with drug doses of 0·3 mg/g 30 min before each radiation fraction. Such treatment gave almost identical enhancement values for both drugs indicating that also after fractionated treatment the effect of the 2 sensitizers appears to be similar when relatively small drug doses are used (Table III).

### *Drug cytotoxicity*

Finally, the potential hypoxic cell

TABLE III.—*Radiosensitizing effect in fractionated treatment*

| Treatment | $TCD_{50}$ (Gy) | ER |
|---|---|---|
| Five daily fractions of radiation alone (control) | 62·1 (57·7–66·9) | — |
| Five fractions of radiation 0·3 mg/g MISO before each fraction | 47·0 (43·7–50·5) | 1·32 (1·10–1·58) |
| Five fractions of radiation 0·3 mg/g nimorazole before each fraction | 49·1 (43·7–55·2) | 1·26 (1·01–1·58) |

Numbers in parentheses represent 95% confidence limits.

TABLE IV.—*Potential cytotoxic effect of MISO and nimorazole*

| Treatment | $TCD_{50}$ (Gy) | ER |
|---|---|---|
| Radiation alone | 56·2 (54·5–57·9) | — |
| MISO 1·0 mg/g after radiation | 55·3 (50·4–60·7) | 1·02 (0·96–1·06) |
| Nimorazole 1·0 mg/g after radiation | 56·0 (50·4–62·4) | 1·00 (0·94–1·08) |

Numbers in parentheses represent 95% confidence limits.

cytotoxicity was estimated by giving the drugs immediately *after* a single dose of irradiation. As seen in Table IV, none of the drugs given in single doses of 1 mg/g was able significantly to alter the $TCD_{50}$ values when compared to irradiation alone. An important drug cytotoxicity against hypoxic tumour cells seems therefore not to occur after such treatment in the present tumour system.

### DISCUSSION

The present study has shown that nimorazole enhances the radiation response in an experimental mammary carcinoma but with an unusual lack of dose–response relationship over a wide dose range. This contrasts with MISO, which has a relatively steep dose–response relationship with ERs in the same range as those observed in similar or other tumour models (Denekamp *et al.*, 1982; Fowler *et al.*, 1976). This lack of dose–response relationship may be the reason why nimorazole has not previously been regarded as a hypoxic sensitizer with clinical potential. However, in the dose range which is relevant to clinical treatment schedules ($\leqq$ 0·3 mg/g per fraction, depending on fractionation scheme) nimorazole produces the same enhancement as MISO. The difference between the 2 drugs is only significant at high concentrations, but these have little interest for clinical radiotherapy since the doses required cannot be achieved in humans without excessive toxicity. The flat dose–response curve may also explain why nimorazole has not been considered to be an effective sensitizer, since a common *in vitro* screening procedure is to establish the dose required to produce an ER of 1·6 (Adams *et al.*, 1979*b*). A similar plateau of the dose–response curve observed in the present study has also been reported using V79 cells *in vitro* (Adams *et al.*, 1979*a*; Midander & Littbrand, submitted for publication).

The difference in dose–response relationship could not be explained by different tumour drug concentrations, although the 2 drugs differ in pharmacokinetics and in lipophilicity. MISO is less lipophilic and has a shorter half-life in mice than nimorazole. However, neither the difference in lipophilicity nor in half-life seems to influence the tumour/plasma ratio in mice (Workman, 1980; Workman & Brown, 1981). The difference in lipophilicity means a greater metabolic degradation of nimorazole (Giraldi *et al.*, 1971), whereas MISO is excreted to a higher degree unmetabolized in the urine (Workman, 1980). The possibility exists that the metabolism of nimorazole results in secondary products which may also act as hypoxic radiosensitizers and that these in turn may contribute to the overall ER. However, preliminary pharmacokinetic analyses of these products have shown that they are not present in significant amounts in either plasma or tumour at the time of irradiation.

Both the plasma concentration, the tumour/plasma ratio, the $LD_{50}$ values and the ERs obtained with MISO are similar to those observed in other studies (Denekamp *et al.*, 1982; Fowler *et al.*, 1976; Rofstad & Brustad, 1978; Workman, 1979). This indicates that the present experimental system is valid for this kind of experiment, and makes the results directly comparable with those of others.

The difference between the toxicity of the 2 drugs which is observed in different experimental systems is a typical example of the problems relating to toxicity evaluation of potential hypoxic radiosensitizers. Thus, nimorazole is probably (though not statistically) more toxic in mice with slightly smaller $LD_{50}$ values. However, MISO has considerably more aerobic toxicity in V79 cells (Adams *et al.*, 1979*b*). Likewise, the $LD_{50}$ values in rats are apparently smaller for MISO than for nimorazole (1680 and 3180 mg/kg respectively after oral administration). Furthermore, comparable data indicate that nimorazole has considerably less acute or chronic toxicity in dogs, and nimorazole seems to be even less toxic in dogs than metronidazole (Roche, unpublished data; FarmItalia, Carlo Erba, unpublished data). In humans nimorazole has been given in doses up to 25 g in 10 days without causing peripheral neuropathy or other severe side effects (Overgaard *et al.*, 1983). Thus nimorazole seems to be considerably better tolerated than MISO, and on the basis of large-animal toxicity it may be tolerated as well as metronidazole (Flagyl) which can be given in daily doses of 6 g/m$^2$ and in total doses up to 100 g (Kapp *et al.*, 1982; Karim, 1978; Urtasun *et al.*, 1975). Therefore it is likely that nimorazole could be given in normal radiation fractionation schedules in doses corresponding to those necessary to produce ERs of ~1·4 in the present tumour after single-dose radiation in air.

Evidently nimorazole with its flat dose–response curve does not promise a greater radiosensitizing effect than MISO, even when given in higher doses. However, if the lack of significant toxicity can be established, it might be a more acceptable drug with a better therapeutic ratio in clinical treatment. Further studies to explore the potential of nimorazole as a hypoxic radiosensitizer in clinical radiotherapy have therefore been started (Overgaard *et al.*, 1983).

We wish to thank Ms Inger Marie Thuesen and Ms Inger Marie Johansen for enthusiastic and skilful technical help, and Ms Lisa Wagner for secretarial assistance.

This work was supported by the Danish Cancer Society. grant no. 24/79 and "Grosserer Sigurd Abrahamson og hustru Addie Abrahamson's mindelegat".

### REFERENCES

Adams, G. E., Clarke, E. D., Flockhart, I. R. & 5 others (1979*a*) Structure–activity relationships in the development of hypoxic cell radiosensitizers. I. Sensitization efficiency. *Int. J. Radiat. Biol.*, **35**, 133.

Adams, G. E., Clarke, E. D., Gray, P. & 4 others (1979*b*) Structure–activity relationships in the development of hypoxic cell radiosensitizers. II. Cytotoxicity and therapeutic ratio. *Int. J. Radiat. Biol.*, **35**, 151.

Brown, J. M. & Lee, W. W. (1980) Pharmacokinetic considerations in radiosensitizer development. In *Radiation Sensitizers.* New York: Masson Publ. USA, Inc., p. 2.

Brown, J. M. & Workman, P. (1980) Partition coefficient as a guide to the development of radiosensitizers which are less toxic than misonidazole. *Radiat. Res.*, **82**, 171.

Brown, J. M., Yu, N. Y. & Workman, P. (1979) Pharmacokinetic considerations in testing hypoxic cell radiosensitizers in mouse tumours. *Br. J. Cancer*, **39**, 310.

Coleman, C. N., Wasserman, T. H., Philips, T. L. & 5 others (1982) Initial pharmacology and toxicology of intravenous desmethylmisonidazole. *Int. J. Radiat. Oncol. Biol. Phys.*, **8**, 371.

Conroy, P. J., McNeill, T. H., Passalacqua, W., Merritt, J., Reich, K. R. & Walker, S. (1982) Nitroimidazole neurotoxicity: are mouse studies predictive? *Int. J. Radiat. Oncol. Biol. Phys.*, **8**, 799.

Denekamp, J., Michael, B. D., Minchinton, A. I., & 4 others (1982) Comparative studies of hypoxic-cell radiosensitization using artificially hypoxic skin *in vivo*. *Br. J. Cancer*, **45**, 247.

Dische, S., Fowler, J. F., Saunders, M. I. & 3 others (1980) A drug for improved radiosensitization in radiotherapy. *Br. J. Cancer*, **42**, 153.

Dische, S., Saunders, M. I., Anderson, P., Stratford, M. R. L. & Minchinton, A. I. (1982) Clinical experience with nitroimidazole as radiosensitizers. *Int. J. Radiat. Oncol. Biol. Phys.*, **8**, 335.

Dische, S., Saunders, M. I., Flockhart, I. R., Lee, M. E. & Anderson, P. (1979) Misonidazole—a drug for trial in radiotherapy and oncology. *Int. J. Radiat. Oncol. Biol. Phys.*, **5**, 851.

Fazekas, J. T., Goodman, R. L. & McLean, C. J. (1981) The value of adjuvant misonidazole in the definitive irradiation of advanced head and neck squamous cancer: an RTOG pilot study (No. 78-02). *Int. J. Radiat. Oncol. Biol. Phys.*, **7**, 1703.

Fowler, J. F., Adams, G. E. & Denekamp, J. (1976) Radiosensitizers of hypoxic cells in solid tumours. *Cancer Treat. Rev.*, **3**, 227.

Giraldi, P. N., Tosolini, G. P., Dradi, E. & 5 others (1971) Studies on antiprotozoans—III. Isolation, identification and quantitative determination in humans of the metabolites of a new trichomonacidal agent. *Biochem. Pharmacol.*, **20**, 339.

Kapp, D. S., Wagner, F. C. & Lawrence, R. (1982) Glioblastoma multiforme: treatment by large dose fraction irradiation and metronidazole. *Int. J. Radiat. Oncol. Biol. Phys.*, **8**, 35.

Karim, A. B. M. F. (1978) Prolonged metronidazole administration with protracted radiotherapy: a pilot study on response of advanced tumours. *Br. J. Cancer*, **37**, (Suppl. III), 299.

Kogelnik, H. D. (1980) Clinical experience with misonidazole. High dose fractions *versus* daily low doses. *Cancer Clin. Trials*, **3**, 179.

Overgaard, J. (1980*a*) Effect of misonidazole and hyperthermia on the radiosensitivity on a C3H mouse mammary carcinoma and its surrounding normal tissue. *Br. J. Cancer*, **41**, 10.

Overgaard, J. (1980*b*) Simultaneous and sequential hyperthermia and radiation treatment of an experimental tumor and its surrounding normal tissue *in vivo*. *Int. Radiat. Oncol. Biol. Phys.*, **6**, 1507.

Overgaard, J., Andersen, A. P., Jensen, R. H. & 6 others (1982) Misonidazole combined with split-course radiotherapy in the treatment of invasive carcinoma of the larynx and the pharynx. *Acta Otolaryngol.*, **386**, (Suppl.) 215.
OVERGAARD, M., OVERGAARD, J. & TIMOTHY, A. R. (1983) Preliminary studies of the pharmacokinetic propreties of nimorazole. *Br. J. Cancer*, (submitted for publication).
PHILLIPS, T. L., WASSERMAN, T. H., JOHNSON, R. J., LEVIN, V. A. & VANRAALTE, G. (1981) Final report on the United States phase I clinical trial of the hypoxic cell radiosensitizers, misonidazole (Ro-07-0582; NSC No. 261037). *Cancer*, **48**, 1697.
ROFSTAD, E. K. & BRUSTAD, T. (1978) The radiosensitizing effect of metronidazole and misonidazole (Ro-07-0582) on a human malignant melanoma grown in the athymic mutant nude mouse. *Br. J. Radiol.*, **51**, 381.
SUIT, H. D., SHALEK, R. J. & WETTE, R. (1965) Radiation response of C3H mouse mammary carcinoma evaluated in terms of cellular radiation sensitivity. In *Cellular Radiation Biology*. Baltimore: Williams & Wilkins. p. 514.
URTASUN, R. C., CHAPMAN, J. D., BAND, P., RABIN, H. R., FRYER, C. G. & STURMWIND, J. (1975) Phase 1 study of high-dose metronidazole: a specific *in vivo* and *in vitro* radiosensitizer of hypoxic cells. *Radiology*, **117**, 129.
WORKMAN, P. (1979) Analysis of the basic 5-nitroimidazole nimorazole in blood by reversed-phase high-performance liquid chromatography, and its application to pharmacokinetic studies in individual mice. *J. Chromatogr.*, **163**, 396.
WORKMAN, P. (1980) Pharmacokinetics of hypoxic cell radiosensitizers. *Cancer Clin. Trials*, **3**, 237.
WORKMAN, P., BLEEHEN, N. M. & WILTSHIRE, C. R. (1980) Phenytoin shortens the half-line of the hypoxic cell radiosensitizer misonidazole in man: implications for possible reduced toxicity. *Br. J. Cancer*, **41**, 302.
WORKMAN, P. & BROWN, J. M. (1981) Structure–pharmacokinetic relationships for misonidazole analogues in mice. *Cancer Chemother. Pharmacol.*, **6**, 39.